# Dysbiosis contributes to chronic constipation development *via* regulation of serotonin transporter in the intestine

**DOI:** 10.1038/s41598-017-10835-8

**Published:** 2017-09-04

**Authors:** Hailong Cao, Xiang Liu, Yingying An, Guoqiong Zhou, Yanrong Liu, Mengque Xu, Wenxiao Dong, Sinan Wang, Fang Yan, Kui Jiang, Bangmao Wang

**Affiliations:** 1 Department of Gastroenterology and Hepatology, General Hospital, Tianjin Medical University, Tianjin, China; 2 Division of Gastroenterology, Hepatology and Nutrition, Department of Pediatrics, Vanderbilt University Medical Center, Nashville, Tennessee USA; 3 Tianjin Key Laboratory of Molecular Drug Research, Tianjin International Joint Academy of Biomedicine, Tianjin, China

## Abstract

Chronic constipation is a prevalent functional gastrointestinal disorder accompanied with intestinal dysbiosis. However, causal relationship between dysbiosis and constipation remains poorly understood. Serotonin transporter (SERT) is a transmembrane transport protein which re-uptakes excessive 5-hydroxytryptamine (5-HT) from effective location to terminate its physiological effects and involves in regulating gastrointestinal motility. In this study, fecal microbiota from patients with constipation and healthy controls were transplanted into the antibiotic depletion mice model. The mice which received fecal microbiota from patients with constipation presented a reducing in intestinal peristalsis and abnormal defecation parameters including the frequency of pellet expulsion, fecal weight and fecal water content. After fecal microbiota transplantation, the SERT expression in the colonic tissue was significantly upregulated, and the content of 5-HT was decreased which negatively correlated with the gastrointestinal transit time. Moverover, fecal microbiota from the mice which received fecal microbiota from patients with constipation also upregulated SERT in Caco-2 cells. Besides, this process accompanied with the decreased abundance of *Clostridium*, *Lactobacillus*, *Desulfovibrio*, and *Methylobacterium* and an increased tend of *Bacteroides* and *Akkermansia*, which also involved in the impairment of intestinal barrier after FMT. Taken together, intestinal dysbiosis may upregulate the SERT expression and contribute to the development of chronic constipation.

## Introduction

Chronic constipation is a globally prevalent functional gastrointestinal disorder with the prevalence 2–20%^1–3^. According to Rome IV criteria, the typical symptoms of chronic constipation is difficult, infrequent, or incomplete defecation^4^. The symptoms of constipation are always continuous and repeated, seriously affecting the patient’s physical and mental health and quality of life. Previous studies have found that chronic constipation was related with variety of factors, such as the change of intestinal nerve cells, myopathy, neurotransmitter and dysbiosis^5, 6^. However, the pathogenesis has not been completely elucidated.

Growing evidences showed that patients with chronic constipation accompanied with intestinal dysbiosis^7^. There are about 10^13^~10^14^ micro-organisms existing in the human digestive system. These microorganisms distribute in the different segments in small intestine as well as large intestine, affecting intestinal physiological function and participating in the life activities which are crucial for the host^8, 9^. However, the prolonged residence time of feces in chronic constipation patients may lead to intestinal dysbiosis, which may further affect the intestinal immune function, motility and barrier function^6, 7, 10^. Specifically, studies have found that *Bacteroides* were more abundant in colonic mucosa of patients with chronic constipation, and the populations of *Clostridium* *difficile* and *Bifidobacterium* were significantly increased. In contrast, levels of *Lactobacillus* and *Faecalibacterium prausnitzii* were decreased. Moreover, *Firmicutes* including *Firmicutes-Coprococcus*, *Firmicutes-Faecalibacterium*, *Firmicutes-Lactococcus*, and *Firmicutes-Roseburia* was independently significantly useful for predicting colonic transit^6, 11^. Nevertheless, the causal relationship between dysbiosis and constipation remains poorly understood.

5-hydroxytryptamine (5-HT) is the key neurotransmitter in the brain-gut axis. Most (over 95%) 5-HT in body is secreted by enterochromaffin cells and it plays a important role in gut motility which has been found in many researches. It also associated with gastrointestinal motility disorder and abnormal sensation^12–14^. The plasma level of 5-HT in constipation-type IBS is reduced^15^, while by contrast, the plasma 5-HT level in diarrhea-type irritable bowel syndrome (IBS) patients is increased^16^. Accumulate evidence shows gut microbes can interact with the human host through modulation of 5-HT signaling^17^. Furthermore, pervious research shown that *Clostridia* can modulate 5-HT signalling through production of soluble metabolites that influence 5-HT synthesis, and affect both gastro-intestinal motility through this mechanism^18^. Recent study showed that *Escherichia coli Nissle 1917* can enhance 5-HT bioavailability in gut tissues through interaction with compounds secreted from host^19^. Serotonin transporter (SERT) is the main regulator of extracellular 5-HT availability^20^. SERT is a transmembrane transport protein which is predominantly expressed by essentially all epithelial cells of the intestinal mucosa^21, 22^. It re-uptakes excessive 5-HT from effective location to terminate its physiological effects, and involves in regulating gastrointestinal motility^18, 23^. Interestingly, *Yano JM et al*. reported that intestinal microbiota can change the level of SERT and regulate gastrointestinal function^18^. Some specific bacteria have been reported to regulate the expression of SERT in intestinal epithelial cells, such as *Listeria monocytogenes* ^20^ and *E. coli* ^24^. Lately, clinical studies have shown that intestinal peristalsis slowed down due to overexpression of progesterone receptor affecting SERT-5-HT pathway to regulate circular muscle contraction^25^. Therefore, intestinal abnormal SERT expression contributes to a variety of functional gastrointestinal disorders.

In the present study we found that the mice which received fecal microbiota from patients with constipation had a reduced trend in intestinal peristalsis and abnormal defecation parameters. Besides, this process cause the upregulated SERT expression in the colonic tissues and Caco-2 cells, on the contrary, the content of 5-HT was significantly decreased. Moreover, it accompanied with the decreased abundance of *Clostridium*, *Lactobacillus*, *Desulfovibrio*, and *Methylobacterium* and an increased tend of *Bacteroides* and *Akkermansia*, and caused the damage of intestinal barrier. Thus, intestinal dysbiosis may upregulate the SERT expression and contribute to the development of chronic constipation. This study will provide a new perspective on intestinal microecological treatment for constipation.

## Results

### The mice receiving fecal microbiota from patients with constipation presented abnormal defecation parameters

**Figure 1:**
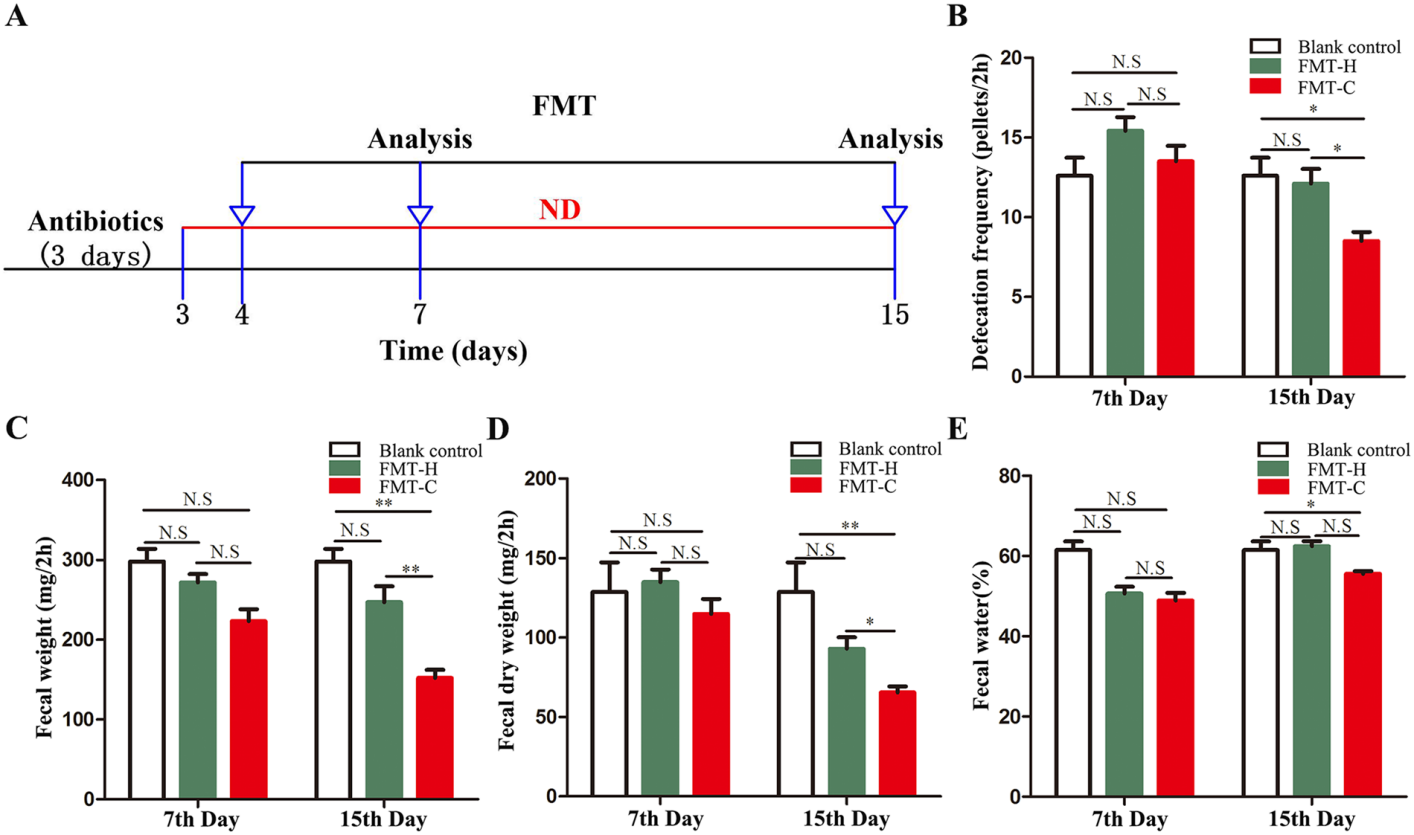
The mice receiving fecal microbiota from patients with constipation presented abnormal defecation parameters. Antibiotic mixture (500 mg of ampicillin, 250 mg of vancomycin, 500 mg neomycin and 250 mg of metronidazole) was given by gavage daily for 3 days before FMT. The mice were inoculated with the fecal microbiota for a total of 7 times over the subsequent 2 weeks (**A**). Parameters of defecation were shown in the blank control, FMT-C group (n = 10), and the FMT-H group (n = 10). In 7^th^ day, frequency of defecation within 2 hours, the changes of fecal weight, dry weight and fecal water within 2 hours in FMT-C group were similar to those in FMT-H group or blank control. While in 15^th^ day, a decrease trend in frequency of defecation within 2 hours, fecal weight, dry weight and fecal water within 2 hours were found in the FMT-C group compared with those in the FMT-H group (**B**–**E**). There is no significance between blank control and FMT-H group in the 7^th^ and 15^th^ day. The parameters on the 15^th^ day have significantly decreased in the FMT-C group compared with those in the FMT-H group and blank control. While there was no significant difference between blank control and FMT-C in the 7^th^ day (**B**–**E**). FMT, fecal microbiota transplantation; FMT-C group, the group that received the fecal microbiota of constipation patients; FMT-H group, the group that received the fecal microbiota of healthy controls; ND, normal diet; N.S, no significance; *P < 0.05, **P < 0.01, ***P < 0.001.

A diagram of our study design is shown in Fig. 1A. We used the parameters of blank control as the baseline data which were treated with antibiotics (defecation pellets in 2 h: 12.6 ± 2.51; fecal weight: 301.8 ± 33.8 mg; fecal dry weight: 119.3 ± 37.2 mg; fecal water: 60.5 ± 10.1%). There is no significance between blank control and FMT-H group in the 7th and 15th day. The parameters on the 15th day have significantly decreased in the FMT-C group compared with blank control. While there was no significant difference between blank control and FMT-C in the 7th day. Meanwhile, the defecation function was evaluated on the 7th day after fecal microbiota transplantation (FMT). The number of defecation pellets in 2 h (FMT-C group: 13.55 ± 3.12 *vs* FMT-H group: 15.42 ± 2.63, *P* > 0.05), the fecal weight (223.2 ± 47.50 mg *vs* 271.86 ± 32.72 mg, *P* > 0.05), fecal dry weight (114.82 ± 29.96 mg *vs* 134.91 ± 25.18 mg, *P* > 0.05) and fecal water content (48.88% ± 6.08% *vs* 50.62% ± 5.42%, *P* > 0.05) in the FMT-C group are decreased, but without significant differences comparing with the FMT-H group. The parameters on the 15th day after FMT showed that the number of defecation pellets in 2 h (FMT-C group: 8.55 ± 1.83 *vs* FMT-H group: 12.14 ± 2.90, *P* < 0.05), the weight of feces (151.90 ± 32.42 mg *vs* 246.72 ± 64.01 mg, *P* < 0.01), fecal dry weight (65.52 ± 11.76 mg *vs* 92.93 ± 23.07 mg, *P* < 0.05) and the fecal water content (56.63% ± 3.01% *vs* 61.95% ± 3.70%, *P* < 0.05) have significantly decreased in the FMT-C group compared with those in the FMT-H group **(**Fig. 1B–E).

### The intestinal dysbiosis contributed to gastrointestinal motility dysfunction of the mice

**Figure 2:**
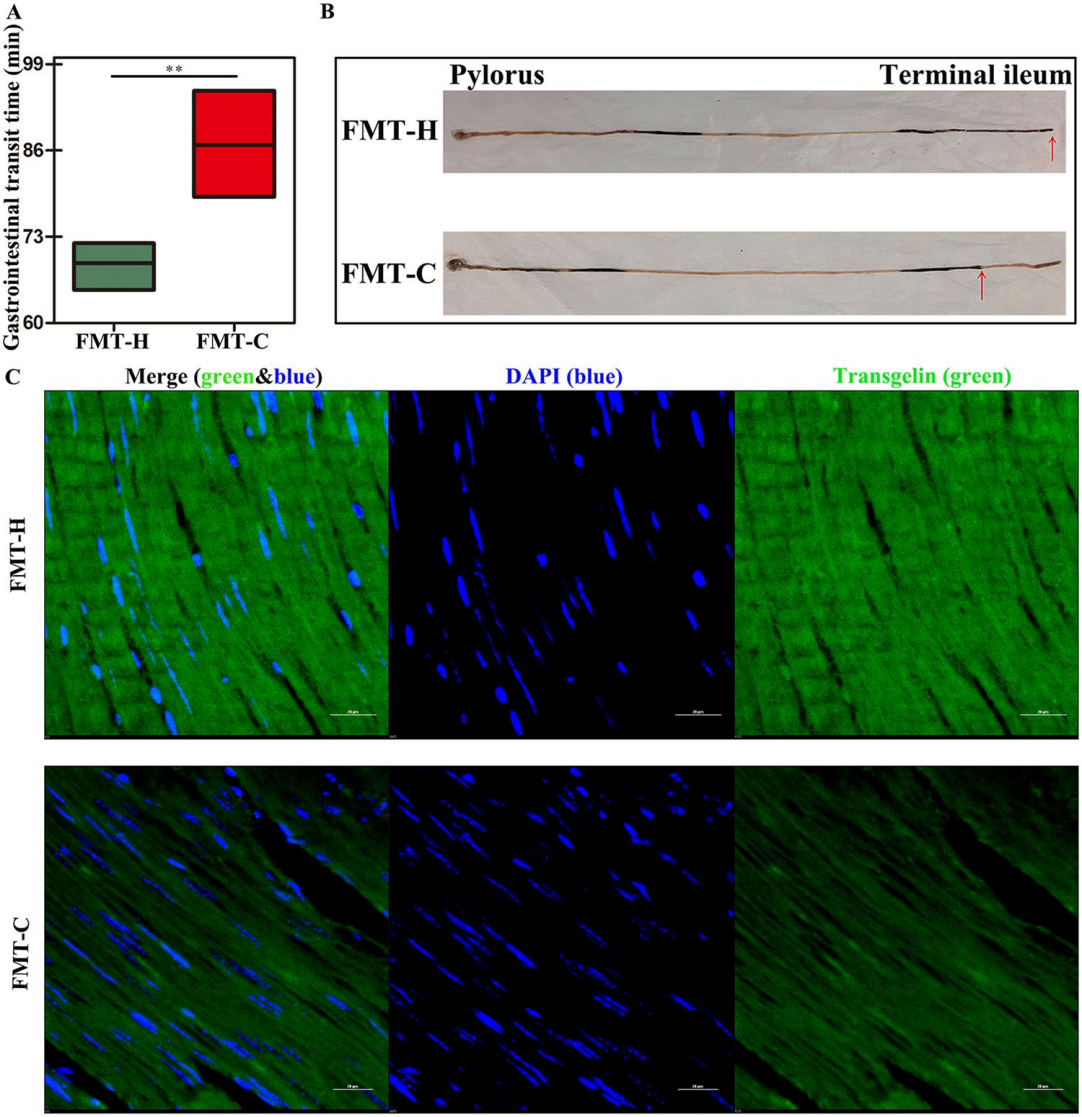
Dysbiosis contributed to gastrointestinal motility dysfunction in mice. GITT was used to evaluate the gastrointestinal motility of mice after fecal microbiota transplantation (FMT). GITT in the FMT-C group was decreased compared with that in the FMT-H group (n = 5) (**A**). Ink propulsion experiment was performed as follow: all of the mice (n = 5) were fasted overnight for 16 h. On the next morning, mice were given ink 0.2 mL/10 g. After a 25 min interval, the mice were killed to collect the segments of stomach to ileocecal junction. Ink propulsion rate (%) = migration distance of ink/whole length of small intestine × 100%. The number of the mice in which the ink reached the ileocecal region was lower in the FMT-C group compared with that in the FMT-H group (40% *vs* 100%*, P* < 0.05) (**B**). Transgelin is an actin stress fibre-associated protein that acts to stabilize actin, which can regulate contractile function of smooth muscle cell. The trangelin expression was decreased in the mice intestinal tissues of FMT-C group, compared with FMT-H group (**C**). Scale bar: 20 μm. GITT, gastrointestinal transient time; FMT-C group, the group that received the fecal microbiota of constipation patients; FMT-H group, the group that received the fecal microbiota of healthy controls. **P < 0.01.

On the 15th day, the gastrointestinal transit time (GITT) was significantly prolonged in the FMT-C group compared with the FMT-H group (83.24 ± 11.31 min *vs* 69.06 ± 2.72 min, *P* < 0.05) **(**Fig. 2A **)**; The percentage of the mice in which the ink reached the ileocecal region in the FMT-C group was decreased compared with that in the FMT-H group (40% *vs* 100%, *P* < 0.05) **(**Fig. 2B **)**.

Transgelin is an actin stress fibre-associated protein that acts to stabilize actin, which is one of the earliest markers of smooth muscle cell differentiation. Transgelin also has functions in regulating contractile function of smooth muscle cell^26^. Transgelin protein levels in the mice intestinal tissues in the present study were decreased in the FMT-C group compared with that in the FMT-H group **(**Fig. 2C **)**. Taken together, these data indicated that the intestinal flora of patients with constipation markedly slowed down the gastrointestinal motility in mice.

### Fecal microbiota of constipation patients upregulated SERT levels in the mice intestinal tissues

**Figure 3:**
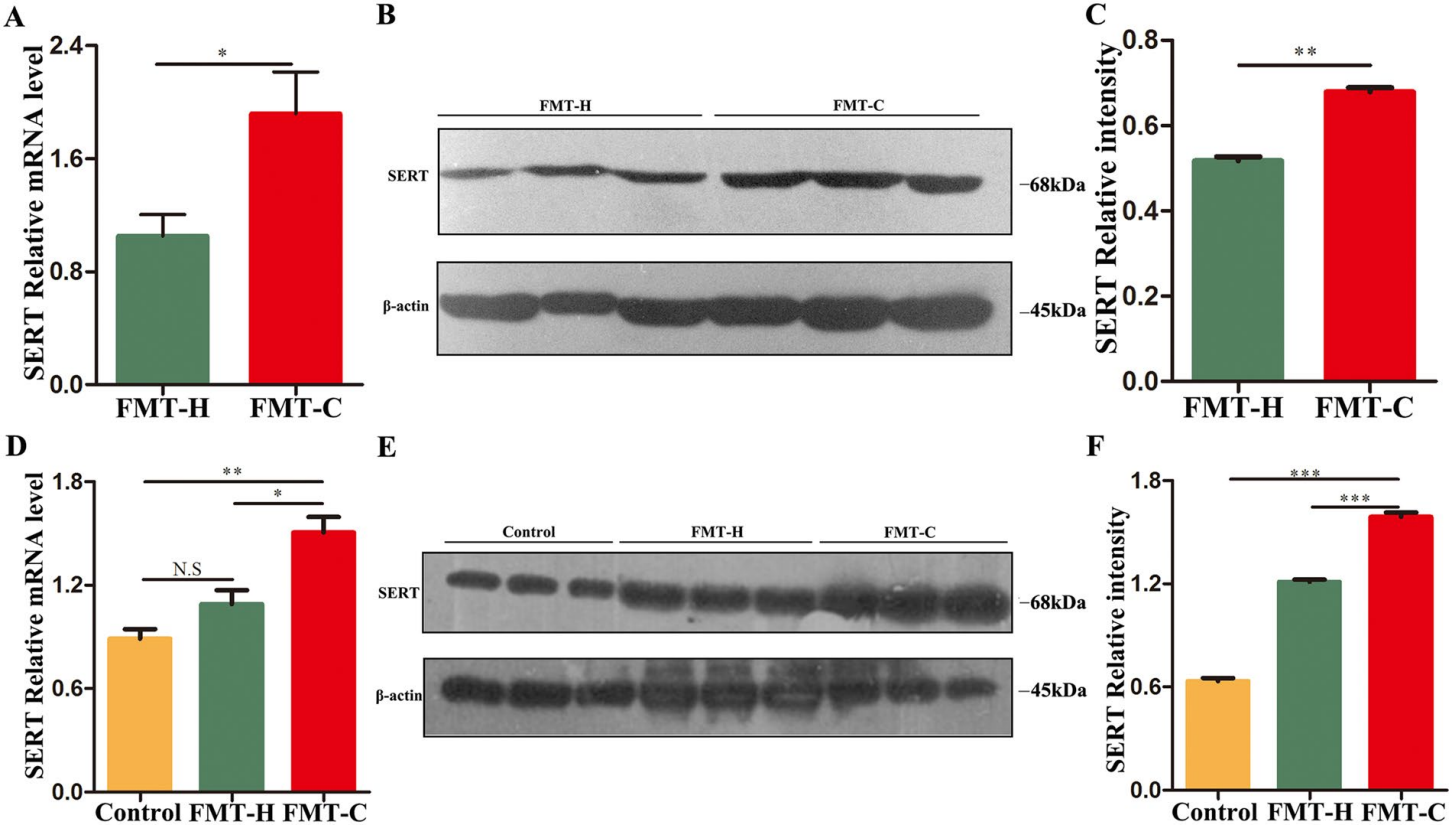
Fecal microbiota of constipation patients upregulated SERT levels in the mice intestinal tissues. Realtime-PCR results showed SERT mRNA level was up-regulated in intestinal tissues of the FMT-C group compared with the FMT-H group (**A**). Intestinal protein levels of SERT in both groups were analyzed by Western blot, using internal control protein β-actin for total protein (**B**). Proteins were quantified by densitometry using an Imaging processor program (Image J) (**C**). Similarly, SERT mRNA levels in Caco-2 cells were detected after the treatment with fecal microbiota from the three groups (Blank control, FMT-H, FMT-C) for 3 h, and the concentration of fecal microbiota was 1 to 2000 (**D**). Protein levels of SERT in Caco-2 cells treated with fecal microbiota from the three groups for 3 h were analyzed by Western blot (**E**). Quantitative analysis of the protein levels of SERT in Caco-2 cells were processed by densitometry with Image J (**F**). The uncropped blots with molecular weights are shown in Fig. S2. FMT-C group, the group that received the fecal microbiota of constipation patients; FMT-H group, the group that received the fecal microbiota of healthy controls; SERT, serotonin transporter; N.S, no significance; *P < 0.05, **P < 0.01, ***P < 0.001.

The SERT mRNA level in colon tissue of the FMT-C group was significantly higher compared with that in the FMT-H group (*P* < 0.05) **(**Fig. 3A **)**, and consistently, Western blot results showed a significant rising trend in SERT protein expression in the FMT-C group **(**Fig. 3B,C **)**.

Next, the fecal bacteria liquid with medium at 1: 2000 was used to stimulate Caco-2 cells. The SERT mRNA level stimulated with fecal liquid from the FMT-C group was significantly higher than that from the FMT-H group and the blank control (*P* < 0.001), while the level of SERT mRNA stimulated with fecal liquid from the FMT-H group was slightly higher compared with blank control group, but without significant difference (*P* > 0.05) **(**Fig. 3D **)**. Western blot results also showed that fecal liquid from the FMT-C group significantly up-regulated SERT protein in Caco-2 cells **(**Fig. 3E,F **)**.

### Fecal microbiota of constipation patients decreased 5-HT levels in the mice intestinal tissues

**Figure 4:**
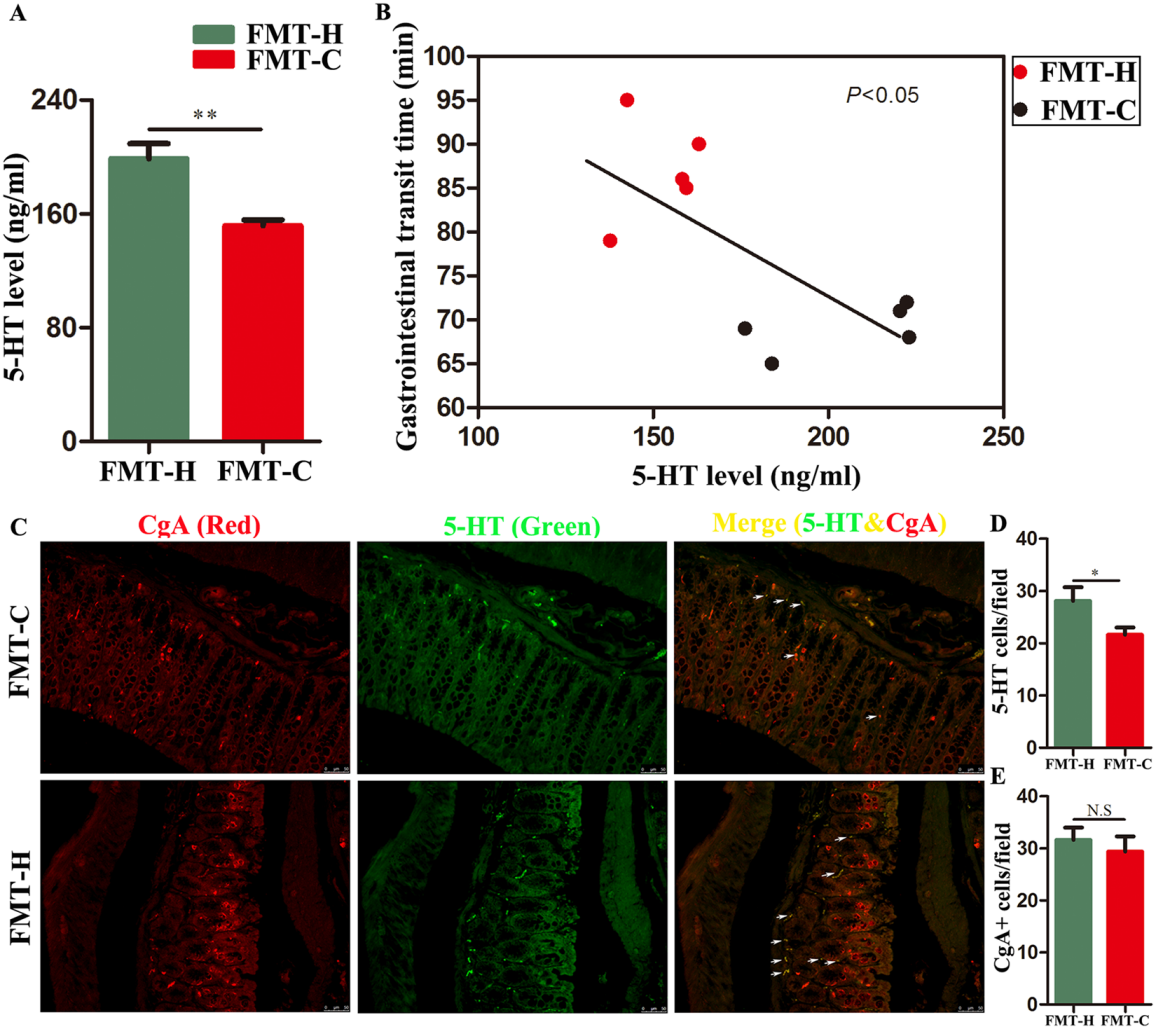
Fecal microbiota of constipation patients decreased 5-HT levels in the mice intestinal tissues. ELISA analysis showed decreased 5-hydroxytryptamine (5-HT) levels in the FMT-C group compared with the FMT-H group (**A**). The 5-HT level was significantly correlated with GITT (**B**). 5-HT secreted by intestinal chromaffin cells. Chromaffin granules protein A (CgA) exists in chromaffin cell secretory granules. Paraffin sections of colonic tissues were immunofluorescence stained with primary antibodies against CgA to mark chromaffin cells (Red) and to mark 5-HT (Green) (**C**). Quantitation of 5-HT+ cell numbers per field of colonic epitheial tissue (**D**). Quantitation of CgA+ cell number per field of colonic epithelial tissue (**E**). FMT-C group, the group that received the fecal microbiota of constipation patients; FMT-H group, the group that received the fecal microbiota of healthy controls; 5-HT, 5-hydroxytryptamine; CgA Chromaffin granules protein A; GITT, gastrointestinal transient time. **P < 0.01. n = 10.

Gut microbiota can affect GI motility by interacting with key cells that regulate GI motility including enteric neurons, glial cells and enterochromaffin cells (ECs)^27, 28^. 5-HT is an important neurotransmitter closely related to the dynamic function of the gastrointestinal tract. ELISA analysis showed that the level of 5-HT in colonic tissue in the FMT-C group was lower than that of the FMT-H group (151.69 ± 10.18 *vs* 198.77 ± 25.99 ng/ml, *P* < 0.01) **(**Fig. 4A **)**. The FMT-C group exhibits a decreased level of colonic 5-HT and an extended GITT compared with those in the FMT-H group, while there is a linear correlation between the level of colonic 5-HT and GITT **(**Fig. 4B **)**. In the GI tract, 5-HT is synthesized by specialized endocrine cells, called ECs, and decreased level of 5-HT in the FMT-C group localized to colonic chromogranin A-positive (CgA+) was found in immunofluorescence **(**Fig. 4C **)**. Less 5-HT cells per field can be found in FMT-C group (Fig. 4D). Furthermore, the number of CgA+cells per field had a rising trend in FMT-H group, however, there is no significant difference between FMT-H group and FMT-C group (Fig. 4E).

### Constipation-induced dysbiosis could be transmitted to the mice

**Figure 5:**
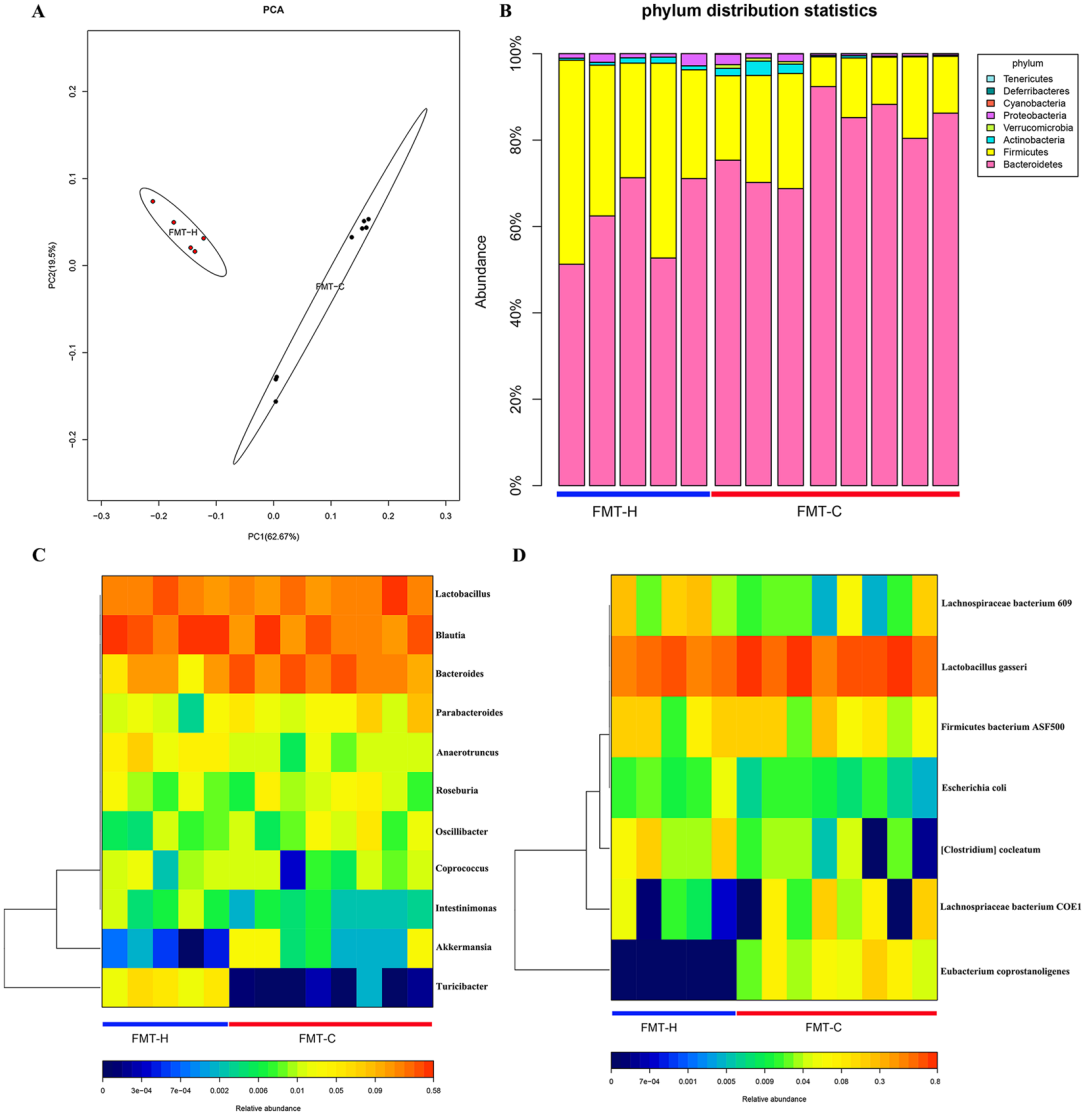
Constipation-induced dysbiosis could be transmitted to the mice. PCA focused on fecal bacterial communities using principal components in two distinct groups. The spatial distance showed the similarity degree of bacterial taxon among samples. FMT-H group: red dots, n = 5; FMT-C group: blue dots; n = 8 (**A**). The phylum level in the FMT-H and FMT-C groups after the 2 weeks experiment (**B**). Heatmap results showed the significant difference in abundance between FMT-H and FMT-C groups at important genus and species levels that after the experiment (**C**,**D**). Different color showed the relative abundance of the community (from cold to warm color means from low to high abundance). PCA, Principal component analysis; FMT-C group, the group that received the fecal microbiota of constipation patients; FMT-H group, the group that received the fecal microbiota of healthy controls.

Pyrosequencing analysis was used to further identify the different components of gut microbiota in mice after FMT. PCA results showed that the fecal microbial community in the FMT-C group was significantly different from that in the FMT-H group. PCA disclosed that the major alteration in the microbial community was promoted by the distinct treatments as shown in Fig. 5A.

Further analysis of microbial phylum showed that the proportion of *Firmicutes* was significantly decreased, whereas *Bacteroidetes* was increased in FMT-C group **(**Fig. 5B **)**. Moreover, analysis at the genus level showed that the relative abundance of *Clostridium*, *Lactobacillus*, *Desulfovibrio* and *Methylobacterium* were significantly lower, while *Bacteroides* and *Akkermansia* have an increased trend in the FMT-C group compared with the FMT-H group **(**Fig. 5C **)**. Furthermore, it also showed the dramatically altered microbiota community at the species level with an increased *Eubacterium-corprostanoligenes*, *Lachnospiraceae-bacterium-COE1* and *Eubacterium coprostanoligenes*, but a downward trend of *Clostridium-leptum, Escherichia coli, Firmicutes bacterium ASF500, Lachnospiraceae bacterium 609* and *Lactobacillus gasseri* in the FMT-C group, as shown in Fig. 5D.

### Fecal microbiota of constipation patients disrupted the intestinal barrier function of the mice

**Figure 6:**
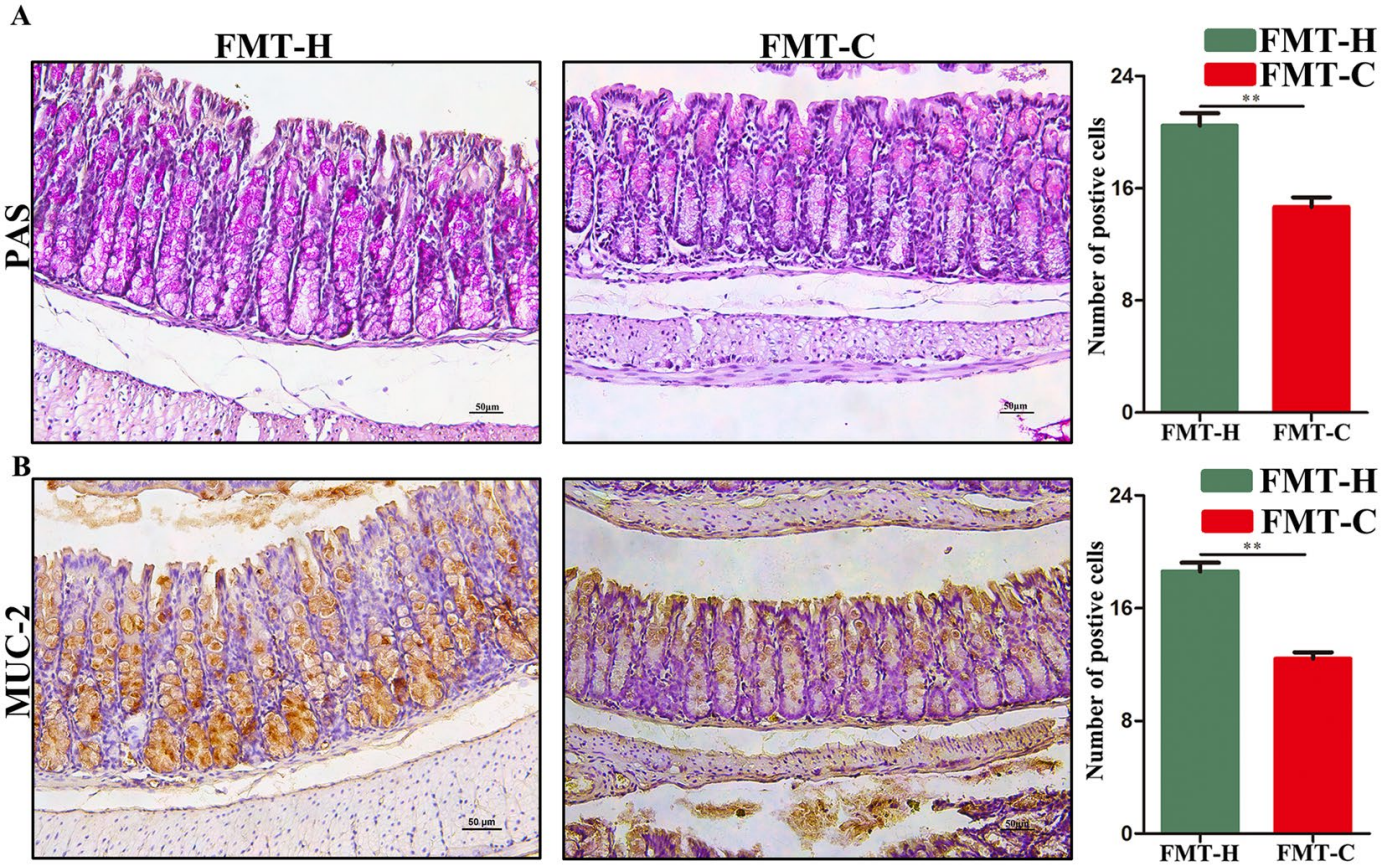
Fecal microbiota of constipation patients disrupted the intestinal barrier function of the mice. PAS staining showed that the average number of PAS positive cells in each crypt of the colon in the FMT-C group was significantly lower than that in the FMT-H group (**A**). MUC2 immunohistochemistry showed the average number of MUC2-positive cells in the crypts of the mice in the colon in two groups (**B**). Scale bar: 50 μm. FMT-C group, the group that received the fecal microbiota of constipation patients; FMT-H group, the group that received the fecal microbiota of healthy controls. *P < 0.05, **P < 0.01, n = 10.

Goblet cells play a vital role in maintaining the thick of mucus layer to defend the pathobionts. It is well known that *Akkermansia* uses mucus as a nutrient source, and is considered as a mucin-degrading bacterium. Based on the pyrosequencing analysis, the relative abundance of *Akkermansia* in the FMT-C group was significantly increased. Accordingly, next we detected the number of mucin-producing goblet cells in the colon from the two groups. PAS staining showed that the average number of goblet cells in each crypt of the colon in the FMT-C group was significantly decreased than that in the FMT-H group (14.44 ± 1.68 *vs* 22.88 ± 0.79, *P* < 0.01) **(**Fig. 6A **)**. MUC2 is the prominent component in the gut secreted from goblet cells. Both the mRNA expression of colonic MUC2 **(**Figure S1 **)** and the average number of MUC2-positive cells in the crypts of the mice (7.49 ± 0.33 *vs* 17.19 ± 0.26, *P* < 0.05) in the FMT-C group was significantly decreased compared with those in the FMT-H group **(**Fig. 6B **)**. These data suggested that constipation-induced dysbiosis could reduce the intestinal goblet cell and destroy the intestinal barrier function.

## Discussion

Chronic constipation is a prevalent functional gastrointestinal disorder worldwide and can lead to significant impairment of quality of life, and also impose considerable costs to society^29^. The gut microbiota plays an important role in many aspects in the health-promoting functions such as metabolic activities, source of energy biogenesis, immune system development, preventing growth of pathogenic bacteria, synthesis of neurotransmitters and neurologic signaling^30, 31^. Chronic constipation often accompanied with intestinal dysbiosis. However, the causal relationship between intestinal dysbiosis and constipation remains poorly understood. In the present study, we found that the mice which received fecal microbiota from patients with constipation presented a reducing in intestinal peristalsis and abnormal defecation parameters. After FMT, the SERT expression in colonic tissue was significantly upregulated, while the content of 5-HT was decreased. This process accompanied with the major alteration in the microbial community promoted by distinct treatments, and the impairment of intestinal barrier. Taken together, intestinal dysbiosis may upregulate the SERT expression and contribute to the development of chronic constipation. These results highlighted the role of intestinal dysbiosis as a potential causative factor in chronic constipation through their impact on the host.

The pathogenesis of constipation is mainly focused on dysfunction of gastrointestinal motility^32, 33^. In recent years, the 5-HT expression of colonic mucosa was reported to be decreased in chronic constipation patients, suggesting that 5-HT may play an important role in the pathogenesis of constipation^15^. In the present study, we found that the mice in FMT-C group showed lower level of 5-HT. It is well known that the majority of 5-HT is produced by ECs and released into the intestinal lumen and blood, and then bind 5-HT receptor to play its role^34^. SERT is a transmembrane protein in the intestinal epithelium, which has a 5-HT transport function. Studies have found that the level of SERT in the IBS was significantly higher than the control group^22^, and SERT could weaken the intestinal circular muscle contraction activity and inhibit intestinal motility to lead to chronic constipation^25^. We also found that the SERT expression in the colonic tissue was significantly upregulated after FMT, which suggested intestinal dysbosis might increase the 5-HT re-uptake by increasing the intestinal SERT level and change the bowel function of the mice.

Previous studies have investigated the intestinal dysbiosis in chronic constipation^6, 35, 36^. Patients with chronic constipation have significantly lower abundance of *Bifidobacterium* and *Lactobacillus* bacteria, and more pathogenic bacteria or fungi^37^. Colonizing of intestinal microbiota to germ-free rats showed that *Lactobacillus acidophilus* and *Bifidobacterium bifidum* could reduce the migrating myoelectric complex period and accelerate small intestinal transit, while *Micrococcus luteus* and *Escherichia coli* showed an inhibitory effect^11^. Our study revealed that the alteration of intestinal microbiota decreased intestinal motility in the FMT-C group, suggesting that intestinal dysbiosis was at least one of the important reasons of the occurrence of chronic constipation. These findings further supports that intestinal microbiota is the potential risk factors for constipation, and probiotic treatment could potentially ameliorate chronic constipation^38–40^.

One of the most important findings was the level of *Akkermansia* was higher in the mice which received fecal microbiota from patients with constipation. *Akkermansia* is a gram-negative anaerobe belonging to *Verrucomicrobia*. *Akkermansia* can produce a large number of enzymes with mucoid degradation^41^, and degrade intestinal mucins, the highly glycosylated proteins of epithelial mucus layer, as its sole source of carbon and nitrogen^42^. Oral administration of *Akkermansia* can improve blood glucose and fat metabolism to remit the metabolic diseases (such as obesity and type 2 diabetes). *Akkermansia* can significantly decrease the Treg population and reduce the pro-inflammatory factor expression to relieve the ulcerative colitis^43–45^. However, until now, few studies have investigated the relationship between *Akkermansia* and chronic constipation. In our study, pyrosequencing analysis showed that the mice in FMT-C group were rich in the *Akkmermansia*, and *Akkmermansia* can degrade intestinal mucin leading to dry stool and finally the impairment of intestinal mucosal barrier, but the mechanisms still need further study. This finding was in accordance with the role of *Akkermansia* in colorectal cancer in the mice^46^. Moreover, the proportion of *Verrucomicrobia* increased in colon cancer patients, and the level of *Akkermansia* increased about 4 times compared with the control group^47^. Thus, *Akkermansia* may possibly be a key connection between constipation and colorectal cancer, which still needs to be further clarified.

In addition, some studies reported that chronic constipation was related with variety of factors, such as circumferential stretch and mechanical stimulation of the mucosa^48, 49^. Meanwhile, the interesting research firstly showed that increased intraluminal pressure could simultaneously release 5-HT from EC cells and initiate the reflexes of intestinal peristalsis *in vivo* and *in vitro* ^50^. Furthermore, mucosal stimulation enhanced the peristaltic reflexes activated by circumferential stretch. These observations implied that intrinsic primary afferent neurons, which were in the myenteric and submucosa plexus, were activated by mucosal 5-HT. Future studies will be more concerned about mucosal stimulation of the large bowel/anus elicit a response.

Meanwhile, colonic migrating motor complexes (CMMCs) play an important role on the motility of intestine. Some studies showed that CMMCs was absent when the colon was empty, and if fecal content was present, the CMMCs frequency would increase^51^. Originally, it was shown that cyclical generation of CMMCs required the release of 5-HT from the mucosa, since antagonists of 5-HT receptors could abolish CMMCs. Our results showed that fecal microbiota of constipation patients decreased 5-HT levels in the mice intestinal tissues. Meanwhile, the mice receiving fecal microbiota from patients with constipation presented abnormal defecation parameters and abnormal intestinal motility. As mentioned above, CMMCs might have a change between FMT-C group and FMT-H group. However, we could not detect the CMMCs at the present time. This was the other limitation of our experiment.

In conclusion, our data suggest that gut dysbiosis could upregulate the expression of intestinal SERT in the intestine, and then increase the uptake and resolution of intestinal 5-HT to inhibit the intestinal motility. It provides a perspective view to uncover the pathogenesis underlying constipation as well as revealing the need for innovative microbiota-mediated therapy for chronic constipation.

## Materials and Methods

### Subject recruitment and sample collection

Patients with chronic constipation and healthy controls were conducted according to Rome III criteria to recruit. Briefly, with onset of symptoms at least 6 months before the diagnosis, and symptoms were consistent with the following diagnostic criteria in the recent 3 months: (1) Two or more of the following conditions must be included: straining; passage of lumpy or hard stools; sensation of incomplete evacuation; sensation of anorectal obstruction; manual maneuvers needed to facilitate defecations; less than three times of defecation every week. (2) Rarely loose stools without laxatives. (3) Inconformity of the diagnostic criteria of irritable bowel syndrome^52^. Slow transit constipation (STC) is diagnosed according to colon transmission test. All the patients took 20 medical barium sulfate diluted in porridge and erect position abdominal X-ray photographs were then taken at 4, 8, 12, 24, 48 hours after meal^53^. Patients with STC are found to retain two or more markers after ingestion 48 h, so taking an abdominal X-ray after 48 h is sufficient to make a confirmation in patients with 48 h. Detailed information including age, sex, defecation frequency, and Bristol score of the subjects were collected in Table 1. Healthy controls are the health physical examination personnel without any diseases. All procedures were performed in accordance with the National Institutes of Health Guide for the Care and Use of Laboratory Animals (NIH Publication No. 80-23) which revised in 1996. Meanwhile, the protocols were reviewed and approved by Ethical Committee of General Hospital, Tianjin Medical University, China. Written informed consent was obtained from all participants and their families.

**Table 1:** Detailed information of the donors.

	Age (years)	Sex (male/female)	Defecation time (times per week)	Bristol score (level)	Colon transmission test
Healthy (n = 5)	18~40	3/2	6~7	3~4	Normal (<2 markers)
STC (n = 5)	27~54	2/3	0~2	1~2	Abnormal (≥2 markers)

Slow transit constipation, STC.

Fresh fecal samples were collected from the constipation patients (n = 5) and healthy controls (n = 5) for FMT. The fecal samples collected from each group were mixed to form fecal liquid at room temperature, and the procedures of preparing the fecal samples for FMT were performed as described in the previous studies^54^. Briefly, fecal samples were dealt with relatively aseptic conditions. Each fecal sample (1 g) was suspended with 10 ml sterile phosphate-buffered saline. The suspensions were filtered through filters with pore diameters of 2.0, 1.0, 0.5 and 0.25 mm. After homogenization and centrifugation, fecal suspensions were obtained and stored in a refrigerator at −80 °C.

### Mice and treatment

C57BL/6 mice were purchased from Beijing Animal Research Center, China, weighting 16–18 g, 6 weeks of age. The mice were fed normal mouse-chow diet under specific pathogen free (SPF) condition. Mice were randomized into two groups: FMT-C group (transplant fecal microbiota of chronic constipation patients, n = 10) and FMT-H group (transplant fecal microbiota of healthy controls, n = 10). The mice were given a mixture of 500 mg of ampicillin, 250 mg of vancomycin, 500 mg neomycin and 250 mg of metronidazole (Sigma-Aldrich, St. Louis, MO, USA) daily for 3 days by gavage, considering as the antibiotic depletion model^55^. The suspensions from fecal sample (0.2 mL/10 g body weight) were transplanted to the mice by gavage. According the previous reports, the mice were inoculated once daily for 3 consecutive days, then on alternate days for 4 times, with a total of 7 times during transfer experiments^56–58^ **(**Fig. 1A **)**. Signs of illness were monitored and body weight was recorded daily. The mice were sacrificed on the 15th day, and colon tissues were collected and stored in 10% formalin solution or −80 °C refrigerator. The animal experiments followed the National Institutes of Health Guide for the Care and Use of Laboratory Animals (NIH Publication No. 80-23), revised in 1996. Animal protocols were approved by the Institutional Animal Care and Use Committee at Tianjin Medical University, Tianjin, P. R. China.

### Cell culture and treatment

The Caco-2 cells as the human intestinal epithelial cells, were grown in Dulbecco’s Modified Eagle Medium (DMEM) media supplemented with 10% fetal bovine serum, 1.0% nonessential amino acid and 1% solution of antimycotic mixture at 37 °C and in air plus 5% CO_2_. Cells were grown as standard monolayers on six-well plate until they reached approximately 70–80% confluency^59^. Cells were serum starved (0.5%) at 37 °C for approximately 12 h before the experiments and then treated with fecal bacteria liquid from FMT-C and FMT-H groups (fecal bacteria liquid -to-cell media ratio: 1:2000) for 3 h according to the previous study^55^, and the blank serum was used as the blank control.

### Defecation function of the mice

Mice in each group were fed in the single cages, we detected the frequency of pellet expulsion, fecal weight, fecal dry weight and fecal water content after giving antibiotics, we used the data of parameters as blank controls. Then the mice were fasted for 16 hours on the 6^th^ and 14^th^ day of the experiment. Freely feeding mice were observed for 2 hours, and the frequency of pellet expulsion and fecal weight were determined. Fecal water content (%) was measured by comparing the weight of the pellets at the end of the experiment and after drying (24 hours at 37 °C). Water content (%) = (wet weight − dry weight)/wet weight × 100% according to the previous studies^60, 61^.

### Gastrointestinal transit time

GITT is the time it takes for food to leave the stomach and travel through the intestines. In our study, five mice in each group were randomly selected for testing GITT and the other five mice were sacrificed for the small intestine advancement test. On the 15th day of experiment, five mice in each group were randomly selected in the single cages. The mice were fasted for 16 hours before experiment, and fed with Indian ink (0.2 mL/10 g body weight-average molar mass) the next day. After that, the time for expulsion of the first blue pellet was determined^62^. Five other mice were subjected to the small intestine advancement test. The mice were also fasted overnight for 16 h and fed with ink. After a 25 min interval, the mice were killed and the segments from stomach to ileocecal junction were collected. Ink propulsion rate (%) = migration distance of ink/whole length of small intestine × 100%^63^.

### Periodic acid schiff (PAS)

The distal colon was removed from all the mice. The tissue was flushed with PBS, fixed in 4% formaldehyde overnight at room temperature and paraffin-embedded. The specimens were then cut into 5 μm sections and stained with Alcian blue periodic acid–Schiff (PAS) (the goblet cells were stained red). The results were expressed as the number of goblet cells per intestinal villus.

### Immunohistochemistry and Immunofluorescence

Formalin-fixed tissues were dehydrated and embedded in paraffin. After being embedded, tissues were sectioned into 4 μm slices which were stained with primary antibodies, rabbit monoclonal anti-MUC2 (1:250, Santa Cruz, USA) overnight at 4 °C. The biotinylated anti-rabbit secondary antibody was stained with horseradish peroxidase (HRP)-streptavidin solution. Finally the sections were counterstained with hematoxylin. Five random areas from a single section were checked for the percentage of positive cells by an independent blinded pathologist. Data were quantified by calculating the average percentages of positive cells in each mouse as the positive rate of cells.

Formalin-fixed tissues were processed for immunofluorescence to evaluate the expression of 5-HT and transgelin protein. Tissues were incubated with primary antibodies (overnight, 4 °C): Part of transgelin, anti-transgelin protein antibody (SM22-alpha-antibody-ab14106) (green, Southern Biotech) was added to the tissue sections for 18 hours at 4 °C. An Alexa Fluor® 488-conjugated goat anti-rabbit IgG polyclonal was used as the secondary antibody(1:5000, Abcam, USA). DAPI (4,6-diamidino-2-phenylindole, blue, Southern Biotech) was lastly applied on the sections. Part of 5-HT, goat anti-mouse 5-HT polyclonal primary antibody(1:500, Abcam, USA) were added to the tissue sections, and then rabbit anti-mouse Chromogranin A polyclonal antibody(1:5000, Abcam, USA) were used. Each of the sections were washed three times with 1 × PBS for 5 minutes and incubated 60 minutes with fluorochrome-conjugated secondary antibodies (5-HT, green; CgA, red) diluted to 2 μg/mL in PBS in the dark. DAPI (4,6-diamidino-2-phenylindole, blue, Southern Biotech) was lastly applied on the sections. We observed and photographed with a fluorescence microscope (Lycra, Germany) for 5-HT and confocal microscopy for transgelin (Nikon, Japan). For 5-HT and CgA staining, numbers of positively-stained puncta were scored blindly, normalized to a field of intestinal mucosa using Image-Pro Plus software, and then averaged across biological replicates.

### ELISA analysis

The 5-HT levels were detected in supernatant of colon tissue homogenates by ELISA according to the manufacturer’s instructions. Appropriate amount of mouse colon tissue was through homogenate, centrifugation to get the supernatant. Samples wells and blank wells were set up to be measured. Meanwhile the three repeat wells were set up. The absorbance at 450 nm (OD value) was measured. The linear regression equation was calculated according to the concentration of the standard and the corresponding OD value of the standard curve, and then the sample OD value of the corresponding 5-HT concentration (ng/ml) was calculated.

### Real-time PCR analysis

Total RNA of the intestinal tissues or Caco-2 cells was extracted using the RNeasy mini kit (TIANGEN, Carlsbad, CA, USA), and cDNA reverse transcription was applied for using the TIANScript RT Kit (TIANGEN, Inc. Beijing, China) according to the manufacturer’s instructions. The Oligonucleotide primers for target genes were shown as follows: GAPDH, (glyceraldehyde-3-phosphate dehydrogenase, 5′-AGGTCGGTGTGAACGGATTTG-3′ and 5′-TGTAGACCATGTAGTTGAGGTCA-3′), SERT (5′-TGG GCG CTC TAC TAC CTC AT -3′ and 5′-ATGTTGTCCTGGGCGAAGTA-3′) and MUC2 (5′-TCGCCCAAGTCGACA CTCA-3′ and 5′-GCAAATAGCCATAGTACAGTTACACAGC-3′). The 2^−ΔΔCt^ was used to calculate relative mRNA expression.

### Western blot analysis

The lysates from the colon tissues or Caco-2 cells were solubilized using RIPA buffer supplemented with protease inhibitors (Solarbio, Beijing, China) and homogenized. And then protein was electrophoresed on a 10% Tris gel with running buffer; then it was blotted to PVDF membrane. Membranes were blocked with nonfat milk and incubated overnight with primary antibodies, anti-SERT antibody (1:1000, Abcam, USA) with anti-β-actin antibody (1:5000, Abcam, USA). Immunoreactive bands were detected after incubation with secondary antibodies (1:5000, EARTHOX, USA). Proteins were quantified densitometrically using Image J software.

### 16 sRNA pyrosequencing analysis

Fecal DNA was extracted from stool samples of the two groups according to the E.Z.N.A method. Stool DNA Kit QIAamp DNA Stool Mini Kit (Omega Bio-Tek, Norcross, GA, USA) following to the manufacturer’s guidelines. Specific primers with barcode targeting V3-V4 hypervariable region of the 16 S rRNA gene were used for PCR amplification in triplicate^64, 65^. The Miseq library was constructed and sequenced on the Illumina 2 × 300 bp MiSeq platform after amplification. The optimizing sequences were usually mapped into operational taxonomic units (OTUs) and picked at 97% similarity in Mothur (version v.1.30.1)^66, 67^. Based on the results of OTUs, community diversity was estimated by Shannon and Simpson index. Similarities were shown by dendrogram among the samples. Principal component analysis (PCA) was carried on the resulting matrix of distances between the two groups.

### Statistical analysis

Statistical analysis was performed on SPSS 22.0 (SPSS, Chicago, IL, USA). Measurement data were expressed as *mean* ± *SD*. Differences were tested by one- way *ANOVA* for paired samples. The results of small intestine advancement test were compared by *Chi-square test* and corrected by *Fisher’s*. *P* value < 0.05 was defined as statistically significant.

## Electronic supplementary material

Supplementary Information
