# Supplementary material for: Dysbiosis contributes to chronic constipation development via regulation of serotonin transporter in the intestine
Source: Sci Rep. 2017 Sep 4;7:10322. doi: 10.1038/s41598-017-10835-8 (PMC5583244; doi:10.1038/s41598-017-10835-8)

**Supplementary Information**

**Dysbiosis contributes to chronic constipation development *via* regulation of serotonin transporter in the intestine**

Hailong Cao^1,2,a^, Xiang Liu ^1,a^, Yingying An^1^, Guoqiong Zhou^1^, Yanrong Liu^3^, Mengque Xu^1^, Wenxiao Dong^1^, Sinan Wang^1^, Fang Yan^1,2^, Kui Jiang^1,*^, and Bangmao Wang^1,*^

**Figure S1. Fecal microbiota of constipation patients downregulated the colonic mRNA expression of MUC2 of the mice.** FMT-C group, the group that received the fecal microbiota of constipation patients; FMT-H group, the group that received the fecal microbiota of healthy controls. *, P< 0.05, n=10.


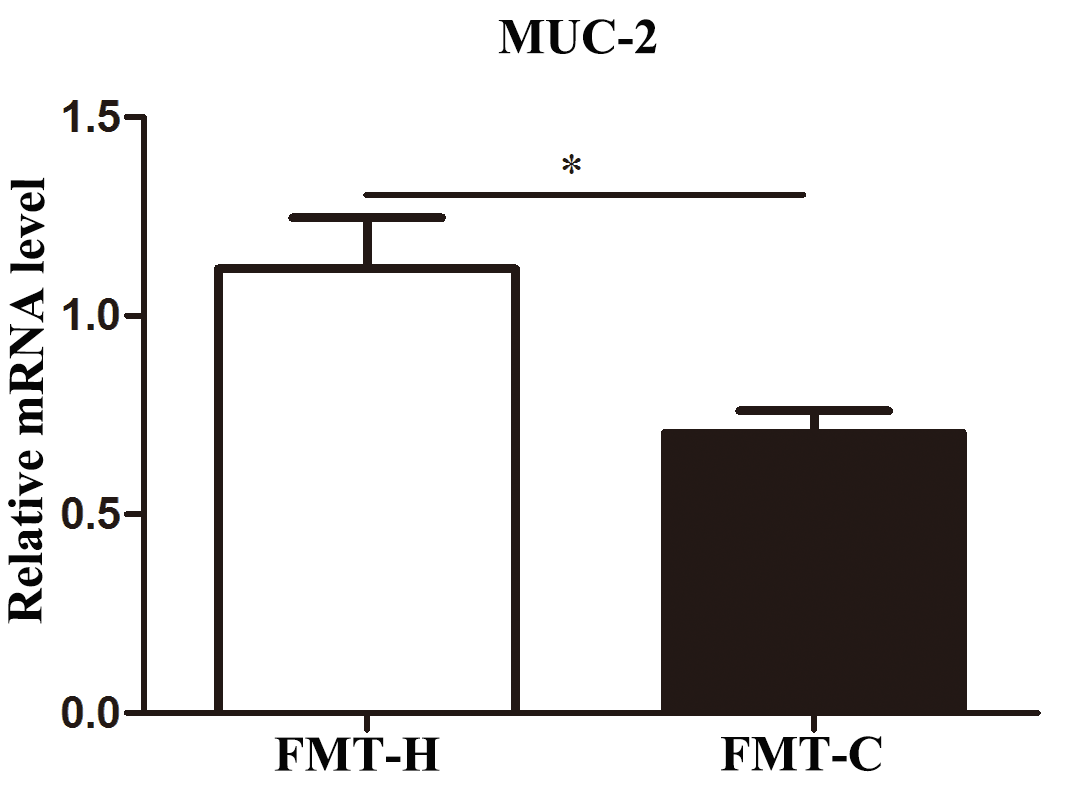


**Figure S2. Original images of blots with molecular weights (KDa).**


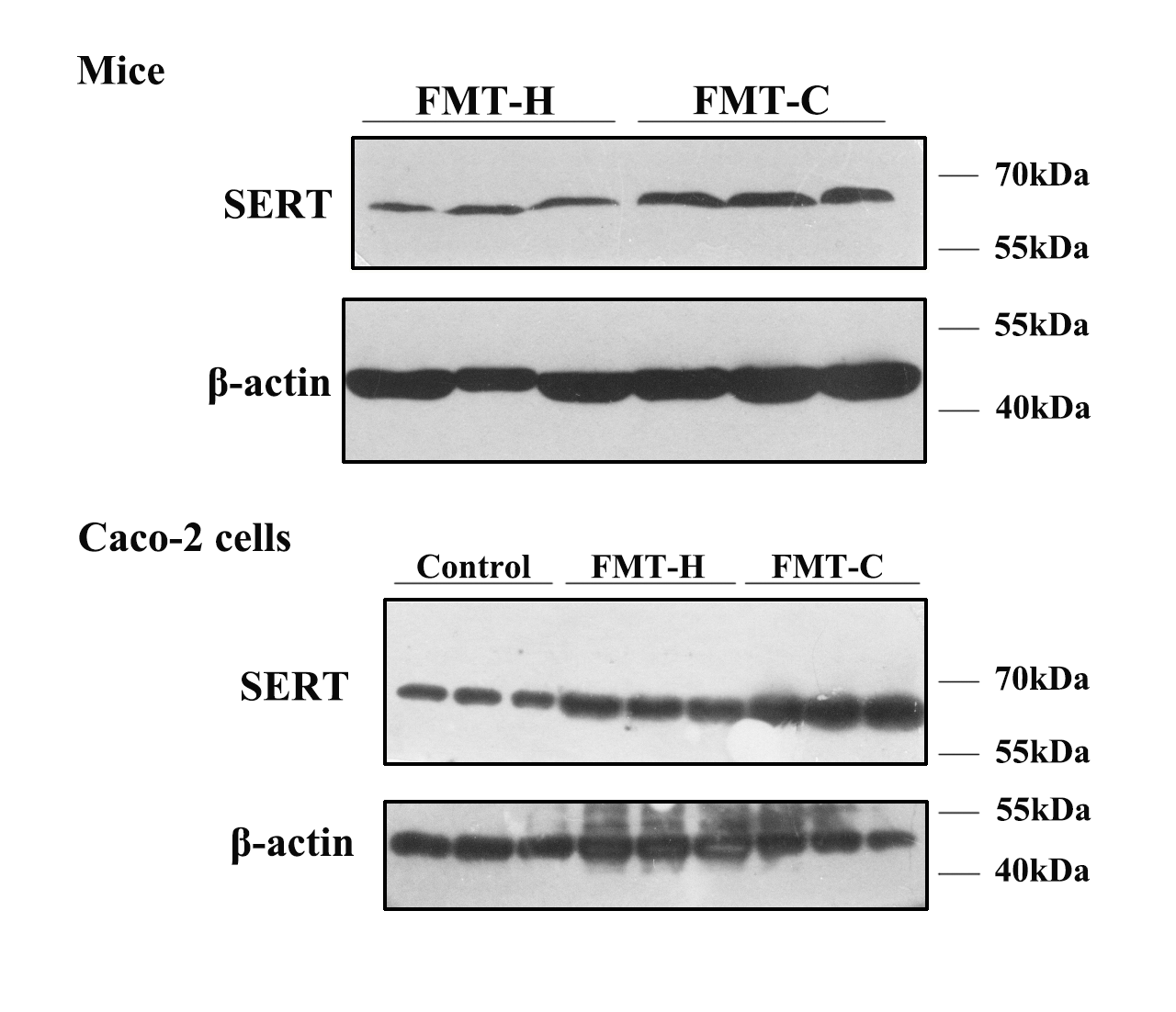

Supplement: Supplementary file 1 — Supplementary Information [file 41598_2017_10835_MOESM1_ESM.doc]
